# 2,4,6-Trimethyl-*N*-[1-(1*H*-pyrrol-2-yl)ethyl­idene]aniline

**DOI:** 10.1107/S1600536812037774

**Published:** 2012-09-08

**Authors:** Bi-yun Su, Lei Li, Jia-Xiang Wang, Xuan-Yan Li

**Affiliations:** a College of Chemistry and Chemical Engineering, Xi’an ShiYou University, Xi’an, Shaanxi 710065, People’s Republic of China

## Abstract

There are two independent mol­ecules in the asymmetric unit of the title compound, C_15_H_18_N_2_, each of which features a *syn* disposition of the N atoms. In each mol­ecule, the pyrrole and benzene rings are essentially perpendicular, with dihedral angles of 78.90 (9) and 79.96 (9)°. In the crystal, the independent mol­ecules are connected by a pair of pyrrole–imino N—H⋯N hydrogen bonds, forming a two-mol­ecule aggregate.

## Related literature

For general background to the imino­pyrrole unit, see: Small *et al.* (1998 ▶); Su *et al.* (2009*a* ▶,*b* ▶); Britovsek *et al.* (2003 ▶); Dawson *et al.* (2000 ▶). For the pyrrole diimine unit, see: Matsuo *et al.* (2001 ▶) and for the pyrrole monoimine unit, see: He *et al.* (2009 ▶).
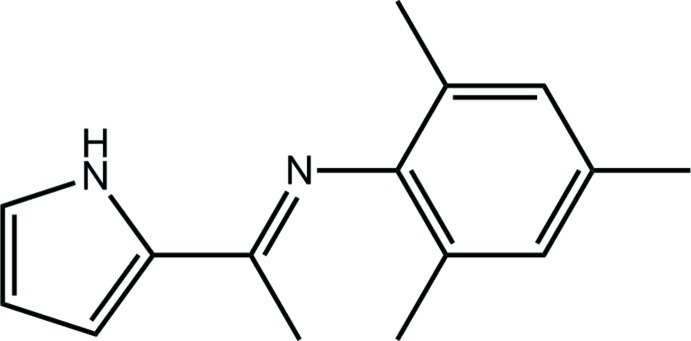


## Experimental

### 

#### Crystal data

C_15_H_18_N_2_
*M* *_r_* = 226.31
Monoclinic, 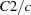

*a* = 29.848 (4) Å
*b* = 7.9668 (11) Å
*c* = 26.325 (4) Å
β = 119.940 (2)°
*V* = 5424.6 (13) Å^3^
*Z* = 16
Mo *K*α radiation
μ = 0.07 mm^−1^
*T* = 296 K
0.37 × 0.24 × 0.18 mm

#### Data collection

Bruker APEXII CCD diffractometer
Absorption correction: multi-scan (*SADABS*; Bruker, 2008 ▶) *T*_min_ = 0.976, *T*_max_ = 0.988
14702 measured reflections
5675 independent reflections
2837 reflections with *I* > 2σ(*I*)
*R*_int_ = 0.054

#### Refinement

*R*[*F* ^2^ > 2σ(*F* ^2^)] = 0.057
*wR*(*F* ^2^) = 0.159
*S* = 0.99
5675 reflections
316 parameters
H-atom parameters constrained
Δρ_max_ = 0.18 e Å^−3^
Δρ_min_ = −0.14 e Å^−3^

### 

Data collection: *APEX2* (Bruker, 2008 ▶); cell refinement: *SAINT* (Bruker, 2008 ▶); data reduction: *SAINT*; program(s) used to solve structure: *SHELXS97* (Sheldrick, 2008 ▶); program(s) used to refine structure: *SHELXL97* (Sheldrick, 2008 ▶); molecular graphics: *SHELXTL* (Sheldrick, 2008 ▶); software used to prepare material for publication: *publCIF* (Westrip, 2010 ▶).

## Supplementary Material

Crystal structure: contains datablock(s) I, global. DOI: 10.1107/S1600536812037774/tk5142sup1.cif

Structure factors: contains datablock(s) I. DOI: 10.1107/S1600536812037774/tk5142Isup2.hkl

Supplementary material file. DOI: 10.1107/S1600536812037774/tk5142Isup3.cml

Additional supplementary materials:  crystallographic information; 3D view; checkCIF report

## Figures and Tables

**Table 1:** Hydrogen-bond geometry (Å, °)

*D*—H⋯*A*	*D*—H	H⋯*A*	*D*⋯*A*	*D*—H⋯*A*
N1—H1⋯N4	0.86	2.23	3.029 (3)	154
N3—H3⋯N2	0.86	2.28	3.060 (3)	151

**Fig. 1.**
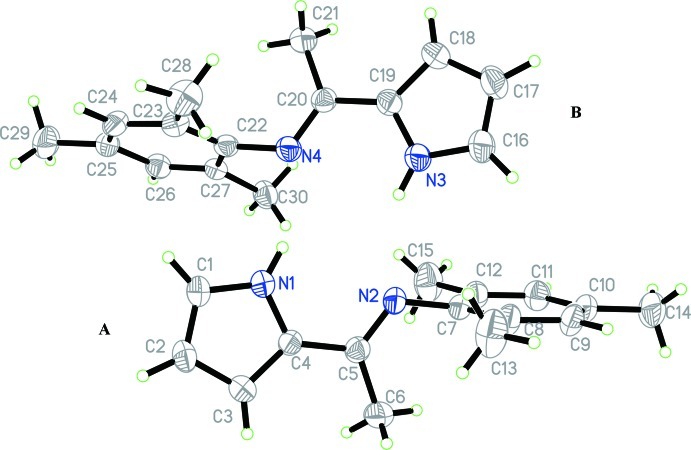
Two independent molecules in the asymmetric unit of the title compound showing the atomic numbering scheme. Displacement ellipsoids are drawn at 30% probability level. H atoms are presented as a small spheres of arbitrary radius.

**Fig. 2.**
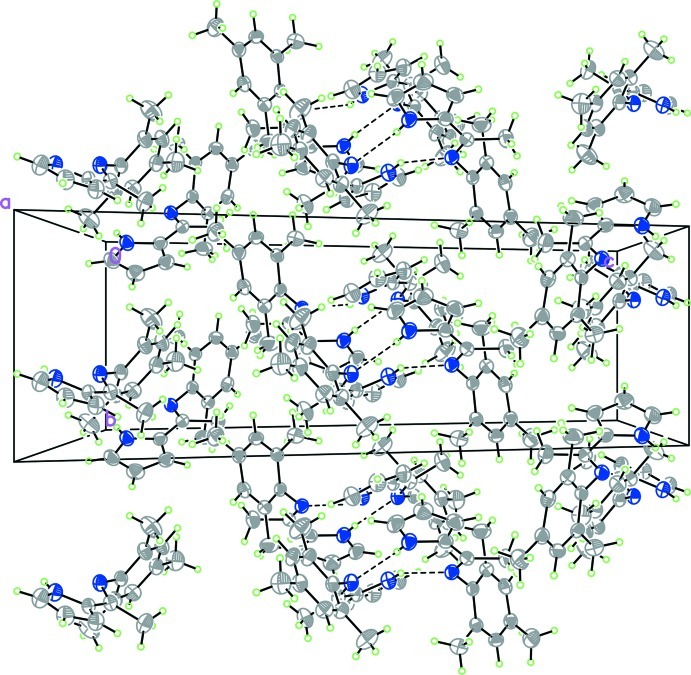
Molecular packing of the title compound with hydrogen bonding shown as dashed lines.

## supplementary crystallographic information

### Comment

Recently, bis(imino)pyridine incorporated late transition metal catalysts have attracted much attention for their antioxidant properties and outstanding activities for olefin polymerization (Small *et al.*, 1998; Su *et al.*, 2009**a*,b*). As a five-membered analogue of the pyridine ring (Matsuo *et al.*, 2001; He *et al.*, 2009), pyrrole has been frequently introduced into the skeleton of bis(imino)pyridine ligands to design new ligands and corresponding metal complexes (Britovsek *et al.*, 2003; Dawson *et al.*, 2000). Bis(imino)pyrrole is usually prepared from Schiff base condensation of 2,5-diacetylpyrrole and an aromatic amine (Matsuo *et al.*, 2001). As a contribution to this research field, we present herein the synthesis of mono(imino)pyrrole from 2-acetyl pyrrole and 2,4,6-trimethylaniline, as well as the crystal structure of the title compound 2,4,6-trimethyl-*N*-[1-(1*H*-pyrrol-2-yl)ethylidene]aniline.

The asymmetric unit of the title compound (Fig. 1) comprises of two crystallographically independent molecules *A* and *B*. These two molecules are connected by a pair of nearly equal N(pyrrole)—H···N(imino) hydrogen bonds, Table 1. In each molecule the pyrrole ring and benzene ring are essentially perpendicular, with dihedral angles of 78.90 (9)° and 79.96 (9)°, respectively. The pyrrole rings of the molecules *A* and *B* present a nearly parallel spatial arrangement with a dihedral angle of 34.70 (11)°, and the benzene rings of the two molecules show a dihedral angle of 29.35 (13)°. Although the two molecules in the asymmetric unit are similar some minor differences in corresponding bond angles are evident, most notably C—N(imino)—C of 118.86 (19) and 120.2 (2)°, for *A* and *B*, respectively.

The crystal packing is stabilized by N—H···N hydrogen bonds (Table 1, Fig. 2) occurring between the independent molecules comprising the asymmetric unit.

### Experimental

The reagents 2-acetyl pyrrole (0.1968 g, 1.80 mmol) and 2,4,6-trimethylaniline (0.2638 g, 1.80 mmol) were placed in a 50 ml flask. After a few drops of acetic acid were added, the mixture was subjected to radiation in a 800 W microwave oven for 3 min and 2 min on a medium–heat setting. The reaction was monitored by *TLC*, and the crude product was purified by silica gel column chromatography (eluant: petroleum ether/ethyl acetate, 5:1 *v/v*). The colourless crystals of the title compound were obtained by recrystallization from ethanol (yield 0.085 g, 20.9%). *M*.pt: 401.4–407.6 K. IR (KBr): ν_C__═__N_ 1646 cm^-1^. ^1^H NMR (400 MHz, CDCl_3_): *δ* 7.16 (s, 2H, phenyl-H), 6.84 (t, 1H, pyrrole-H), 6.63 (t, 1H, pyrrole-H), 6.19 (d, 1H, pyrrole-H), 2.23 (s, 3H, –N=C(CH_3_)), 1.94 (d, 9H, phenyl-CH_3_). MS (EI): m/*z* 225 (*M*). Anal. Calcd for C_15_H_18_N_2_: C, 79.61; H, 8.02; N, 12.38. Found: C, 79.71; H, 7.362; N, 12.39.

### Refinement

All H atoms were placed at calculated positions and refined as riding, with C—H = 0.93–0.96 Å, N—H = 0.86 Å, and with *U*_iso_(H) = 1.2 *U*_eq_(C, N) or 1.5 *U*_eq_(C) for methyl H atoms. In the crystal structure, there is an 33 Å^3^ void, but the low electron density (0.18 e Å^-3^) in the difference Fourier map suggests no solvent molecule occupying this void.

### Crystal data

**Table:**

C_15_H_18_N_2_	*Z* = 16
*M**_r_* = 226.31	*F*(000) = 1952
Monoclinic, *C*2/*c*	*D*_x_ = 1.108 Mg m^−^^3^
Hall symbol: -C 2yc	Mo *K*α radiation, λ = 0.71073 Å
*a* = 29.848 (4) Å	θ = 2.7–26.8°
*b* = 7.9668 (11) Å	µ = 0.07 mm^−^^1^
*c* = 26.325 (4) Å	*T* = 296 K
β = 119.940 (2)°	Block, colourless
*V* = 5424.6 (13) Å^3^	0.37 × 0.24 × 0.18 mm

### Data collection

**Table:**

Bruker APEXII CCD diffractometer	5675 independent reflections
Radiation source: fine-focus sealed tube	2837 reflections with *I* > 2σ(*I*)
Graphite monochromator	*R*_int_ = 0.054
φ and ω scans	θ_max_ = 26.8°, θ_min_ = 2.7°
Absorption correction: multi-scan (*SADABS*; Bruker, 2008)	*h* = −37→37
*T*_min_ = 0.976, *T*_max_ = 0.988	*k* = −10→9
14702 measured reflections	*l* = −29→33

### Refinement

**Table:**

Refinement on *F*^2^	Secondary atom site location: difference Fourier map
Least-squares matrix: full	Hydrogen site location: inferred from neighbouring sites
*R*[*F*^2^ > 2σ(*F*^2^)] = 0.057	H-atom parameters constrained
*wR*(*F*^2^) = 0.159	*w* = 1/[σ^2^(*F*_o_^2^) + (0.060*P*)^2^ + 0.950*P*] where *P* = (*F*_o_^2^ + 2*F*_c_^2^)/3
*S* = 0.99	(Δ/σ)_max_ = 0.028
5675 reflections	Δρ_max_ = 0.18 e Å^−^^3^
316 parameters	Δρ_min_ = −0.14 e Å^−^^3^
0 restraints	Extinction correction: *SHELXL97* (Sheldrick, 2008), Fc^*^=kFc[1+0.001xFc^2^λ^3^/sin(2θ)]^-1/4^
Primary atom site location: structure-invariant direct methods	Extinction coefficient: 0.0019 (3)

### Special details

**Table:**

Geometry. All e.s.d.'s (except the e.s.d. in the dihedral angle between two l.s. planes) are estimated using the full covariance matrix. The cell e.s.d.'s are taken into account individually in the estimation of e.s.d.'s in distances, angles and torsion angles; correlations between e.s.d.'s in cell parameters are only used when they are defined by crystal symmetry. An approximate (isotropic) treatment of cell e.s.d.'s is used for estimating e.s.d.'s involving l.s. planes.
Refinement. Refinement of *F*^2^ against ALL reflections. The weighted *R*-factor *wR* and goodness of fit *S* are based on *F*^2^, conventional *R*-factors *R* are based on *F*, with *F* set to zero for negative *F*^2^. The threshold expression of *F*^2^ > σ(*F*^2^) is used only for calculating *R*-factors(gt) *etc*. and is not relevant to the choice of reflections for refinement. *R*-factors based on *F*^2^ are statistically about twice as large as those based on *F*, and *R*- factors based on ALL data will be even larger.

### Fractional atomic coordinates and isotropic or equivalent isotropic displacement parameters (Å^2^)

**Table:**

	*x*	*y*	*z*	*U*_iso_*/*U*_eq_	
N1	0.17414 (8)	−0.0243 (2)	0.55671 (9)	0.0611 (6)	
H1	0.1455	0.0292	0.5365	0.073*	
N2	0.14363 (7)	0.1397 (2)	0.62945 (8)	0.0530 (5)	
N3	0.03652 (7)	0.2001 (2)	0.52301 (9)	0.0578 (5)	
H3	0.0668	0.1573	0.5437	0.069*	
N4	0.08934 (7)	0.1824 (2)	0.45990 (8)	0.0541 (5)	
C1	0.19777 (11)	−0.1177 (3)	0.53393 (13)	0.0735 (8)	
H1A	0.1860	−0.1334	0.4942	0.088*	
C2	0.24147 (12)	−0.1846 (3)	0.57869 (14)	0.0759 (8)	
H2	0.2647	−0.2551	0.5754	0.091*	
C3	0.24487 (10)	−0.1272 (3)	0.63084 (12)	0.0655 (7)	
H3A	0.2712	−0.1520	0.6686	0.079*	
C4	0.20241 (9)	−0.0274 (3)	0.61649 (11)	0.0523 (6)	
C5	0.18741 (9)	0.0629 (3)	0.65329 (10)	0.0499 (6)	
C6	0.22526 (10)	0.0608 (3)	0.71790 (10)	0.0679 (7)	
H6A	0.2153	0.1429	0.7371	0.102*	
H6B	0.2592	0.0866	0.7246	0.102*	
H6C	0.2255	−0.0485	0.7334	0.102*	
C7	0.12933 (8)	0.2298 (3)	0.66607 (9)	0.0461 (6)	
C8	0.10410 (9)	0.1483 (3)	0.69156 (10)	0.0538 (6)	
C9	0.08898 (9)	0.2413 (3)	0.72524 (10)	0.0576 (7)	
H9	0.0721	0.1869	0.7422	0.069*	
C10	0.09813 (9)	0.4116 (3)	0.73436 (10)	0.0551 (6)	
C11	0.12191 (9)	0.4894 (3)	0.70688 (10)	0.0545 (6)	
H11	0.1281	0.6042	0.7122	0.065*	
C12	0.13682 (8)	0.4037 (3)	0.67175 (10)	0.0460 (6)	
C13	0.09381 (12)	−0.0379 (3)	0.68352 (13)	0.0818 (9)	
H13A	0.0751	−0.0717	0.7027	0.123*	
H13B	0.1261	−0.0973	0.7003	0.123*	
H13C	0.0737	−0.0632	0.6425	0.123*	
C14	0.08290 (12)	0.5089 (3)	0.77271 (13)	0.0853 (9)	
H14A	0.0486	0.4771	0.7635	0.128*	
H14B	0.0837	0.6270	0.7658	0.128*	
H14C	0.1067	0.4845	0.8132	0.128*	
C15	0.16042 (10)	0.4941 (3)	0.64095 (12)	0.0648 (7)	
H15A	0.1586	0.6130	0.6456	0.097*	
H15B	0.1418	0.4664	0.6000	0.097*	
H15C	0.1959	0.4609	0.6575	0.097*	
C16	0.00067 (10)	0.2114 (3)	0.53974 (12)	0.0686 (8)	
H16	0.0047	0.1752	0.5754	0.082*	
C17	−0.04220 (10)	0.2844 (3)	0.49581 (12)	0.0675 (7)	
H17	−0.0729	0.3060	0.4956	0.081*	
C18	−0.03163 (9)	0.3212 (3)	0.45087 (11)	0.0633 (7)	
H18	−0.0539	0.3733	0.4155	0.076*	
C19	0.01769 (9)	0.2664 (3)	0.46857 (10)	0.0477 (6)	
C20	0.04682 (9)	0.2658 (3)	0.43821 (10)	0.0513 (6)	
C21	0.02468 (10)	0.3648 (4)	0.38190 (12)	0.0747 (8)	
H21A	0.0480	0.3599	0.3668	0.112*	
H21B	0.0200	0.4796	0.3894	0.112*	
H21C	−0.0081	0.3180	0.3537	0.112*	
C22	0.11795 (9)	0.1761 (3)	0.42984 (10)	0.0496 (6)	
C23	0.10903 (9)	0.0452 (3)	0.39048 (11)	0.0588 (7)	
C24	0.14020 (10)	0.0325 (3)	0.36587 (11)	0.0628 (7)	
H24	0.1343	−0.0543	0.3396	0.075*	
C25	0.17990 (9)	0.1440 (3)	0.37872 (11)	0.0573 (6)	
C26	0.18833 (10)	0.2692 (3)	0.41837 (12)	0.0631 (7)	
H26	0.2153	0.3441	0.4281	0.076*	
C27	0.15826 (9)	0.2884 (3)	0.44436 (11)	0.0560 (6)	
C28	0.06663 (12)	−0.0811 (4)	0.37605 (15)	0.0992 (11)	
H28A	0.0669	−0.1642	0.3498	0.149*	
H28B	0.0338	−0.0249	0.3577	0.149*	
H28C	0.0722	−0.1344	0.4114	0.149*	
C29	0.21288 (11)	0.1278 (4)	0.35027 (13)	0.0861 (9)	
H29A	0.2454	0.1833	0.3740	0.129*	
H29B	0.1954	0.1786	0.3121	0.129*	
H29C	0.2189	0.0112	0.3466	0.129*	
C30	0.16873 (12)	0.4276 (4)	0.48756 (14)	0.0938 (10)	
H30A	0.1422	0.5113	0.4698	0.141*	
H30B	0.2017	0.4773	0.4989	0.141*	
H30C	0.1690	0.3828	0.5216	0.141*	

### Atomic displacement parameters (Å^2^)

**Table:**

	*U*^11^	*U*^22^	*U*^33^	*U*^12^	*U*^13^	*U*^23^
N1	0.0641 (14)	0.0634 (13)	0.0535 (13)	0.0138 (10)	0.0275 (12)	−0.0060 (11)
N2	0.0544 (13)	0.0537 (12)	0.0478 (12)	0.0072 (10)	0.0231 (10)	−0.0029 (9)
N3	0.0505 (12)	0.0746 (14)	0.0548 (13)	0.0111 (10)	0.0311 (11)	0.0053 (11)
N4	0.0511 (12)	0.0681 (13)	0.0496 (12)	0.0031 (10)	0.0302 (11)	−0.0017 (10)
C1	0.086 (2)	0.0714 (18)	0.0683 (19)	0.0134 (16)	0.0419 (18)	−0.0145 (15)
C2	0.080 (2)	0.0676 (18)	0.091 (2)	0.0182 (15)	0.051 (2)	−0.0020 (17)
C3	0.0669 (17)	0.0607 (16)	0.0685 (18)	0.0152 (14)	0.0334 (15)	0.0070 (14)
C4	0.0565 (15)	0.0469 (14)	0.0550 (16)	0.0057 (11)	0.0290 (13)	0.0022 (12)
C5	0.0534 (15)	0.0474 (13)	0.0476 (14)	0.0027 (12)	0.0243 (13)	0.0019 (11)
C6	0.0625 (17)	0.0840 (19)	0.0529 (16)	0.0136 (14)	0.0256 (14)	0.0061 (14)
C7	0.0441 (13)	0.0492 (14)	0.0386 (13)	0.0055 (11)	0.0157 (11)	−0.0008 (11)
C8	0.0566 (15)	0.0509 (15)	0.0505 (15)	−0.0007 (12)	0.0241 (13)	0.0010 (12)
C9	0.0607 (16)	0.0672 (17)	0.0491 (15)	0.0000 (13)	0.0306 (13)	0.0087 (13)
C10	0.0570 (15)	0.0602 (16)	0.0479 (15)	0.0095 (12)	0.0261 (13)	−0.0007 (12)
C11	0.0569 (15)	0.0461 (14)	0.0578 (16)	0.0020 (11)	0.0266 (14)	−0.0009 (12)
C12	0.0416 (13)	0.0491 (14)	0.0447 (13)	0.0073 (10)	0.0196 (11)	0.0050 (11)
C13	0.102 (2)	0.0559 (17)	0.093 (2)	−0.0117 (16)	0.052 (2)	−0.0010 (16)
C14	0.106 (2)	0.093 (2)	0.074 (2)	0.0111 (18)	0.058 (2)	−0.0090 (17)
C15	0.0612 (16)	0.0644 (17)	0.0787 (19)	0.0026 (13)	0.0422 (16)	0.0043 (14)
C16	0.0639 (18)	0.091 (2)	0.0681 (18)	0.0114 (15)	0.0458 (17)	0.0108 (16)
C17	0.0550 (17)	0.088 (2)	0.0724 (19)	0.0064 (14)	0.0413 (16)	−0.0015 (16)
C18	0.0501 (15)	0.0796 (18)	0.0580 (16)	0.0102 (13)	0.0253 (14)	0.0041 (14)
C19	0.0461 (14)	0.0558 (14)	0.0424 (14)	−0.0003 (11)	0.0231 (12)	−0.0013 (12)
C20	0.0483 (15)	0.0582 (15)	0.0469 (14)	−0.0026 (12)	0.0235 (12)	−0.0047 (12)
C21	0.0650 (18)	0.096 (2)	0.0638 (18)	0.0155 (15)	0.0324 (15)	0.0206 (16)
C22	0.0490 (14)	0.0601 (15)	0.0439 (13)	0.0045 (12)	0.0263 (12)	0.0000 (12)
C23	0.0562 (15)	0.0745 (17)	0.0548 (15)	−0.0115 (13)	0.0345 (14)	−0.0128 (14)
C24	0.0662 (17)	0.0772 (18)	0.0537 (16)	−0.0068 (14)	0.0364 (15)	−0.0142 (14)
C25	0.0545 (16)	0.0723 (17)	0.0547 (16)	0.0036 (13)	0.0344 (14)	0.0014 (14)
C26	0.0566 (16)	0.0676 (17)	0.0726 (18)	−0.0109 (13)	0.0378 (15)	−0.0057 (15)
C27	0.0519 (15)	0.0628 (16)	0.0556 (15)	−0.0032 (12)	0.0284 (13)	−0.0073 (13)
C28	0.098 (2)	0.111 (2)	0.117 (3)	−0.046 (2)	0.074 (2)	−0.049 (2)
C29	0.083 (2)	0.110 (2)	0.093 (2)	−0.0015 (18)	0.064 (2)	0.0007 (19)
C30	0.091 (2)	0.093 (2)	0.108 (3)	−0.0230 (18)	0.058 (2)	−0.042 (2)

### Geometric parameters (Å, º)

**Table:**

N1—C1	1.354 (3)	C14—H14B	0.9600
N1—C4	1.365 (3)	C14—H14C	0.9600
N1—H1	0.8600	C15—H15A	0.9600
N2—C5	1.287 (3)	C15—H15B	0.9600
N2—C7	1.427 (3)	C15—H15C	0.9600
N3—C16	1.348 (3)	C16—C17	1.355 (4)
N3—C19	1.358 (3)	C16—H16	0.9300
N3—H3	0.8600	C17—C18	1.399 (3)
N4—C20	1.286 (3)	C17—H17	0.9300
N4—C22	1.426 (3)	C18—C19	1.375 (3)
C1—C2	1.356 (4)	C18—H18	0.9300
C1—H1A	0.9300	C19—C20	1.445 (3)
C2—C3	1.402 (3)	C20—C21	1.510 (3)
C2—H2	0.9300	C21—H21A	0.9600
C3—C4	1.380 (3)	C21—H21B	0.9600
C3—H3A	0.9300	C21—H21C	0.9600
C4—C5	1.445 (3)	C22—C27	1.391 (3)
C5—C6	1.500 (3)	C22—C23	1.399 (3)
C6—H6A	0.9600	C23—C24	1.377 (3)
C6—H6B	0.9600	C23—C28	1.509 (3)
C6—H6C	0.9600	C24—C25	1.381 (3)
C7—C8	1.394 (3)	C24—H24	0.9300
C7—C12	1.400 (3)	C25—C26	1.374 (3)
C8—C9	1.392 (3)	C25—C29	1.511 (3)
C8—C13	1.508 (3)	C26—C27	1.382 (3)
C9—C10	1.381 (3)	C26—H26	0.9300
C9—H9	0.9300	C27—C30	1.505 (3)
C10—C11	1.387 (3)	C28—H28A	0.9600
C10—C14	1.512 (3)	C28—H28B	0.9600
C11—C12	1.389 (3)	C28—H28C	0.9600
C11—H11	0.9300	C29—H29A	0.9600
C12—C15	1.498 (3)	C29—H29B	0.9600
C13—H13A	0.9600	C29—H29C	0.9600
C13—H13B	0.9600	C30—H30A	0.9600
C13—H13C	0.9600	C30—H30B	0.9600
C14—H14A	0.9600	C30—H30C	0.9600
			
C1—N1—C4	110.0 (2)	H15A—C15—H15B	109.5
C1—N1—H1	125.0	C12—C15—H15C	109.5
C4—N1—H1	125.0	H15A—C15—H15C	109.5
C5—N2—C7	118.86 (19)	H15B—C15—H15C	109.5
C16—N3—C19	109.9 (2)	N3—C16—C17	108.4 (2)
C16—N3—H3	125.0	N3—C16—H16	125.8
C19—N3—H3	125.0	C17—C16—H16	125.8
C20—N4—C22	120.2 (2)	C16—C17—C18	107.2 (2)
N1—C1—C2	108.6 (2)	C16—C17—H17	126.4
N1—C1—H1A	125.7	C18—C17—H17	126.4
C2—C1—H1A	125.7	C19—C18—C17	107.6 (2)
C1—C2—C3	106.9 (2)	C19—C18—H18	126.2
C1—C2—H2	126.5	C17—C18—H18	126.2
C3—C2—H2	126.5	N3—C19—C18	106.88 (19)
C4—C3—C2	108.2 (3)	N3—C19—C20	122.4 (2)
C4—C3—H3A	125.9	C18—C19—C20	130.7 (2)
C2—C3—H3A	125.9	N4—C20—C19	119.3 (2)
N1—C4—C3	106.3 (2)	N4—C20—C21	123.9 (2)
N1—C4—C5	122.9 (2)	C19—C20—C21	116.9 (2)
C3—C4—C5	130.7 (2)	C20—C21—H21A	109.5
N2—C5—C4	119.2 (2)	C20—C21—H21B	109.5
N2—C5—C6	124.4 (2)	H21A—C21—H21B	109.5
C4—C5—C6	116.4 (2)	C20—C21—H21C	109.5
C5—C6—H6A	109.5	H21A—C21—H21C	109.5
C5—C6—H6B	109.5	H21B—C21—H21C	109.5
H6A—C6—H6B	109.5	C27—C22—C23	120.1 (2)
C5—C6—H6C	109.5	C27—C22—N4	120.0 (2)
H6A—C6—H6C	109.5	C23—C22—N4	119.5 (2)
H6B—C6—H6C	109.5	C24—C23—C22	118.7 (2)
C8—C7—C12	120.3 (2)	C24—C23—C28	120.7 (2)
C8—C7—N2	120.5 (2)	C22—C23—C28	120.6 (2)
C12—C7—N2	119.0 (2)	C23—C24—C25	122.4 (2)
C9—C8—C7	118.9 (2)	C23—C24—H24	118.8
C9—C8—C13	120.3 (2)	C25—C24—H24	118.8
C7—C8—C13	120.8 (2)	C26—C25—C24	117.5 (2)
C10—C9—C8	122.3 (2)	C26—C25—C29	121.4 (2)
C10—C9—H9	118.8	C24—C25—C29	121.0 (2)
C8—C9—H9	118.8	C25—C26—C27	122.6 (2)
C9—C10—C11	117.2 (2)	C25—C26—H26	118.7
C9—C10—C14	121.4 (2)	C27—C26—H26	118.7
C11—C10—C14	121.4 (2)	C26—C27—C22	118.6 (2)
C12—C11—C10	123.0 (2)	C26—C27—C30	120.9 (2)
C12—C11—H11	118.5	C22—C27—C30	120.4 (2)
C10—C11—H11	118.5	C23—C28—H28A	109.5
C11—C12—C7	118.1 (2)	C23—C28—H28B	109.5
C11—C12—C15	121.2 (2)	H28A—C28—H28B	109.5
C7—C12—C15	120.8 (2)	C23—C28—H28C	109.5
C8—C13—H13A	109.5	H28A—C28—H28C	109.5
C8—C13—H13B	109.5	H28B—C28—H28C	109.5
H13A—C13—H13B	109.5	C25—C29—H29A	109.5
C8—C13—H13C	109.5	C25—C29—H29B	109.5
H13A—C13—H13C	109.5	H29A—C29—H29B	109.5
H13B—C13—H13C	109.5	C25—C29—H29C	109.5
C10—C14—H14A	109.5	H29A—C29—H29C	109.5
C10—C14—H14B	109.5	H29B—C29—H29C	109.5
H14A—C14—H14B	109.5	C27—C30—H30A	109.5
C10—C14—H14C	109.5	C27—C30—H30B	109.5
H14A—C14—H14C	109.5	H30A—C30—H30B	109.5
H14B—C14—H14C	109.5	C27—C30—H30C	109.5
C12—C15—H15A	109.5	H30A—C30—H30C	109.5
C12—C15—H15B	109.5	H30B—C30—H30C	109.5
			
C4—N1—C1—C2	0.7 (3)	C19—N3—C16—C17	0.5 (3)
N1—C1—C2—C3	−0.9 (3)	N3—C16—C17—C18	−0.9 (3)
C1—C2—C3—C4	0.7 (3)	C16—C17—C18—C19	1.0 (3)
C1—N1—C4—C3	−0.3 (3)	C16—N3—C19—C18	0.1 (3)
C1—N1—C4—C5	179.1 (2)	C16—N3—C19—C20	−177.5 (2)
C2—C3—C4—N1	−0.3 (3)	C17—C18—C19—N3	−0.7 (3)
C2—C3—C4—C5	−179.6 (2)	C17—C18—C19—C20	176.7 (2)
C7—N2—C5—C4	−179.47 (19)	C22—N4—C20—C19	178.2 (2)
C7—N2—C5—C6	0.0 (3)	C22—N4—C20—C21	−1.6 (4)
N1—C4—C5—N2	5.5 (3)	N3—C19—C20—N4	8.6 (4)
C3—C4—C5—N2	−175.4 (2)	C18—C19—C20—N4	−168.4 (2)
N1—C4—C5—C6	−174.0 (2)	N3—C19—C20—C21	−171.6 (2)
C3—C4—C5—C6	5.1 (4)	C18—C19—C20—C21	11.4 (4)
C5—N2—C7—C8	−87.0 (3)	C20—N4—C22—C27	94.9 (3)
C5—N2—C7—C12	98.7 (3)	C20—N4—C22—C23	−92.1 (3)
C12—C7—C8—C9	−3.4 (3)	C27—C22—C23—C24	−1.2 (4)
N2—C7—C8—C9	−177.6 (2)	N4—C22—C23—C24	−174.2 (2)
C12—C7—C8—C13	177.4 (2)	C27—C22—C23—C28	177.8 (3)
N2—C7—C8—C13	3.1 (3)	N4—C22—C23—C28	4.8 (4)
C7—C8—C9—C10	−0.1 (4)	C22—C23—C24—C25	0.1 (4)
C13—C8—C9—C10	179.2 (2)	C28—C23—C24—C25	−178.9 (3)
C8—C9—C10—C11	2.0 (4)	C23—C24—C25—C26	1.1 (4)
C8—C9—C10—C14	−178.0 (2)	C23—C24—C25—C29	−179.1 (2)
C9—C10—C11—C12	−0.5 (3)	C24—C25—C26—C27	−1.2 (4)
C14—C10—C11—C12	179.4 (2)	C29—C25—C26—C27	179.0 (2)
C10—C11—C12—C7	−2.8 (3)	C25—C26—C27—C22	0.2 (4)
C10—C11—C12—C15	177.5 (2)	C25—C26—C27—C30	−179.9 (3)
C8—C7—C12—C11	4.7 (3)	C23—C22—C27—C26	1.1 (4)
N2—C7—C12—C11	179.01 (19)	N4—C22—C27—C26	174.1 (2)
C8—C7—C12—C15	−175.5 (2)	C23—C22—C27—C30	−178.8 (3)
N2—C7—C12—C15	−1.2 (3)	N4—C22—C27—C30	−5.9 (4)

### Hydrogen-bond geometry (Å, º)

**Table:**

*D*—H···*A*	*D*—H	H···*A*	*D*···*A*	*D*—H···*A*
N1—H1···N4	0.86	2.23	3.029 (3)	154
N3—H3···N2	0.86	2.28	3.060 (3)	151
